# Burden of HIV-Related Stigma and Post-Partum Depression: A Cross-Sectional Study of Patients Attending Prevention of Mother-to-Child Transmission Clinic at Kenyatta National Hospital in Nairobi

**DOI:** 10.3389/fpsyt.2020.532557

**Published:** 2021-02-25

**Authors:** Obadia Yator, Muthoni Mathai, Tele Albert, Manasi Kumar

**Affiliations:** ^1^Department of Psychiatry, School of Medicine, College of Health Sciences, University of Nairobi, Nairobi, Kenya; ^2^Other, Nairobi, Kenya; ^3^Department of Psychiatry, College of Health Sciences, University of Nairobi, Nairobi, Kenya

**Keywords:** postpartum depression, internal stigma, HIV related stigma, discrimination and external stigma, prevention of mother to child HIV transmission

## Abstract

**Background:** We look at how various HIV-related stigma subtypes, especially internalizing types, interact with postpartum depression (PPD) among women living with HIV. Additionally, we identify key psychosocial risk factors that influence stigma and PPD among women attending Prevention of Mother-to-Child Transmission (PMTCT) clinics.

**Methods:** In this cross-sectional design, 123 women living with HIV were recruited. Participants ages between 18 and 50, who were at least 8 weeks postpartum seeking PMTCT services at Kenyatta National Hospital (KNH), between June and September 2014 participated in the study. *HIV/AIDS Stigma Instrument—PLWHA (HASI–P)* was used to assesses stigma and Postpartum depression was assessed by *Edinburgh Postnatal Depression Scale (EPDS)*. Bivariate and multivariate regression models were used to determine the individual characteristics associated with the HIV-related stigma Scale. Post survey a few in-depth-interviews were conducted to explore individuals' stigma and depression experiences.

**Results:** The mean age was 31.2 years (SD = 5.2). Fifty-nine (48%) women screened positive for significant depressive symptoms. Post-partum depression was a significant predictor of internalized stigma, enacted, and total stigma (*P* < 0.05). Older age was associated with less internalized stigma. Living with a partner was associated with more internalized stigma. Having an income above 100 USD per month was protective against stigma. Having good family social support was protective against internalized stigma. A higher educational level was protective against enacted stigma. Being treated for STIs was a risk factor for both enacted and overall stigma.

**Conclusions:** HIV-related stigma needs to be addressed through integrated mental health care programs in PMTCT. Postpartum depression requires comprehensive management to improve short- and long-term outcomes of women living with HIV.

## Background

### HIV Burden in Perinatal Women in Sub-Saharan Africa

Several sub-Saharan African (SSA) countries have a high prevalence of Human Immunodeficiency Virus (HIV) among pregnant women and women of childbearing age (1). Although HIV prevalence among the general population has reduced in Kenya, women continue to be disproportionately affected by the epidemic and, as reported in 2014, 7.6% of women were living with HIV compared with 5.6% of men (2). HIV-related stigma and discrimination have become important areas of focus within behavioral research to promote inclusivity and to address social marginalization of affected individuals and their families (3, 4). Acquired Immune Deficiency Syndrome (AIDS) stigma is characterized by, “prejudice, discounting, discrediting, and discrimination directed at people perceived to be living with HIV and the individuals, groups, and communities with which they are associated” (5). HIV-related stigma has also been found to be associated with depression in the general population of persons living with HIV (6, 7). A higher level of HIV-related stigma has been strongly associated with a higher level of depression and a low level of self-efficacy (8).

People living with HIV (PLWH) experience numerous mental and psychological sequela of stigma including, stress, fear, anxiety, decreased self-esteem, and depression (7, 9). Furthermore, pregnant women living with HIV may experience additional stressors including financial hardships (10), reduced social support, and concern for the physical well-being of their children (11, 12). Stigmatized persons may also internalize the beliefs held in the community and develop self-defacing internal representations of themselves (internalized stigma), possibly leading to demoralization, diminished self-efficacy, and emotional distress (13). Self-stigma was reported to be quite potent in a Ugandan study (2011), where participants described themselves as “useless” and the “same as dead” (14). HIV-related stigma has been well-documented to negatively impact quality of life and overall health outcomes among people living with HIV (15).

### Association Between Internalized Stigma and Depression

Lack of women's empowerment, as well as depression, may be critical risk factors for HIV-related stigma and discrimination (16). A finding from Kenya confirmed that women experiencing major depression with an EPDS score of 13 and above at the postpartum visit tended to be more likely to have experienced HIV-related stigma (16). In a Kenyan longitudinal observational study, internalized stigma was found to be a significant predictor of depression in women with high internalized stigma (17). HIV-related internalized stigma results in feelings of low self-worth which was found to be one of the strong predictors of PPD (18).

### Known Risk Factors Associated With Postpartum Depression

People living with HIV have a high prevalence of depression globally (19). Internalized stigma is the endorsement and internalization of negative evaluations held by others (20). Enacted stigma refers to discriminatory behaviors directed toward people with HIV who are viewed as carrying a stigmatized condition (21). Recent studies have shown that PLWH who report experiences of HIV-related stigma also report low levels of perceived social support (22). Having emotionally supportive family and friends may help decrease the perceived legitimacy of negative evaluations of others and help PLWH develop a more positive sense of self, leading to less internalization of stigma (23). Given that stigma is a substantial barrier to accessing HIV care and prevention services, there is a need to understand the dynamics around internalized and enacted stigma in order to improve these services. In this study, our objective was to determine the association between HIV-related stigma subtypes and PPD among women attending PMTCT clinics. In addition, we tried to identify key psychosocial risk factors that influence stigma and PPD. The psychosocial issues affecting women living with HIV are not adequately addressed in the PMTCT program and thus, this study underscores the need for embedding mental health services across all such facilities. Some conjectures about the relationship between depression and internalized stigma have been proposed in this paper. Both internalized stigma (self-stigma being a variant) and depression have a nexus, so we assume that women are depressed because of the stigma they have experienced, or that because of their depression they are more likely to view people's actions negatively and we assume that this is related to their HIV status (17, 24).

## Methods

### Participant Recruitment

In this cross-sectional study, eligible participants who were postnatal women living with HIV aged between 18 and 50 years were recruited using convenience sampling. All postpartum women who were attending the PMTCT at KNH between June and September 2014, who met eligibility criteria and who were willing to participate were enrolled. Our target population was from an urban setting with the majority having better exposure to formal education. All participants provided written informed consent for study participation. The study was approved by the Kenyatta National Hospital/University of Nairobi Ethical and Scientific Research Committee.

## Measures

*A Socio-demographic questionnaire* was used to gather data (that included marital status, age occupation, education level, perceived family social support, monthly income, intimate partner violence, persons residing with them, and being treated for a sexually transmitted infections (STIs) in the past month, or a partner engaging in extramarital affairs).

Severity of depressive symptoms was assessed using the 10-items *Edinburgh Postnatal Depression Scale* (25). EPDS is an internationally validated tool for screening perinatal depression and has previously been used in other studies in sub-Saharan Africa (26). EPDS has been shown to have good test-retest reliabilities as well as good sensitivity for detecting major depression (27). A cut-off of 13 is recommended for probable major depression and a cut-off of 10 is recommended for probable minor depression (28). In our study, we used a cut-off 13 for significant depressive symptoms. This tool has been used in similar studies such as the Kenyan study addressing linkage to HIV care, postpartum depression, and HIV-related stigma in pregnant women that found EPDS had good internal consistency (Cronbach's α = 0.82) (7). EPDS has also been translated and validated into Kiswahili in Kenya (29). An EPDS cut-off score of 13 has been identified as being a marker for significant depressive symptoms (30).

HIV-related stigma was assessed using *HIV/AIDS Stigma Instrument—PLWHA (HASI–P). HASI–P* is a 33-item instrument assessing six subscales of HIV-related stigma (31). HASI-P has six sub-scales each with items inquiring on perceptions toward life in relation to living with HIV. The five items on negative self-perception look at HIV-related stigma within one's self in the form of negative automatic thoughts whereas as the other five sub-scales (verbal abuse-8 items, social isolation-5 items, fear of contagion−6 items, health care neglect-7 items, and workplace stigma-2 items) assess one's perception toward others and the external environment during their day-to-day interaction while living with HIV.

Both internalized and enacted stigma combined, yielded an overall stigma score. Another study from Kenya also used *HASI–P* when addressing linkage to HIV care, postpartum depression, and HIV-related stigma in pregnant women and the HASI-P had good internal consistency of α = 0.87 (16, 17). HASI–P in our study findings had good internal consistency (Cronbach's α = 0.85). Therefore, the reliability of this tool was 93.9%. After administering the sociodemographic questionnaire and assessing HIV-related stigma using HASI-P, qualitative interviews were carried out with six participants: three participants with elevated EPDS scores of >20 and another group of three with low EPDS scores of <13. These participants were interviewed using *semi-structured open-ended questions* exploring their feelings and perceptions of their HIV status in relation to PPD, HIV-related stigma and gauging of available support and quality of their interpersonal relationships.

## Conceptual Frame Work for HIV-Related Stigma

**Figure 1:**
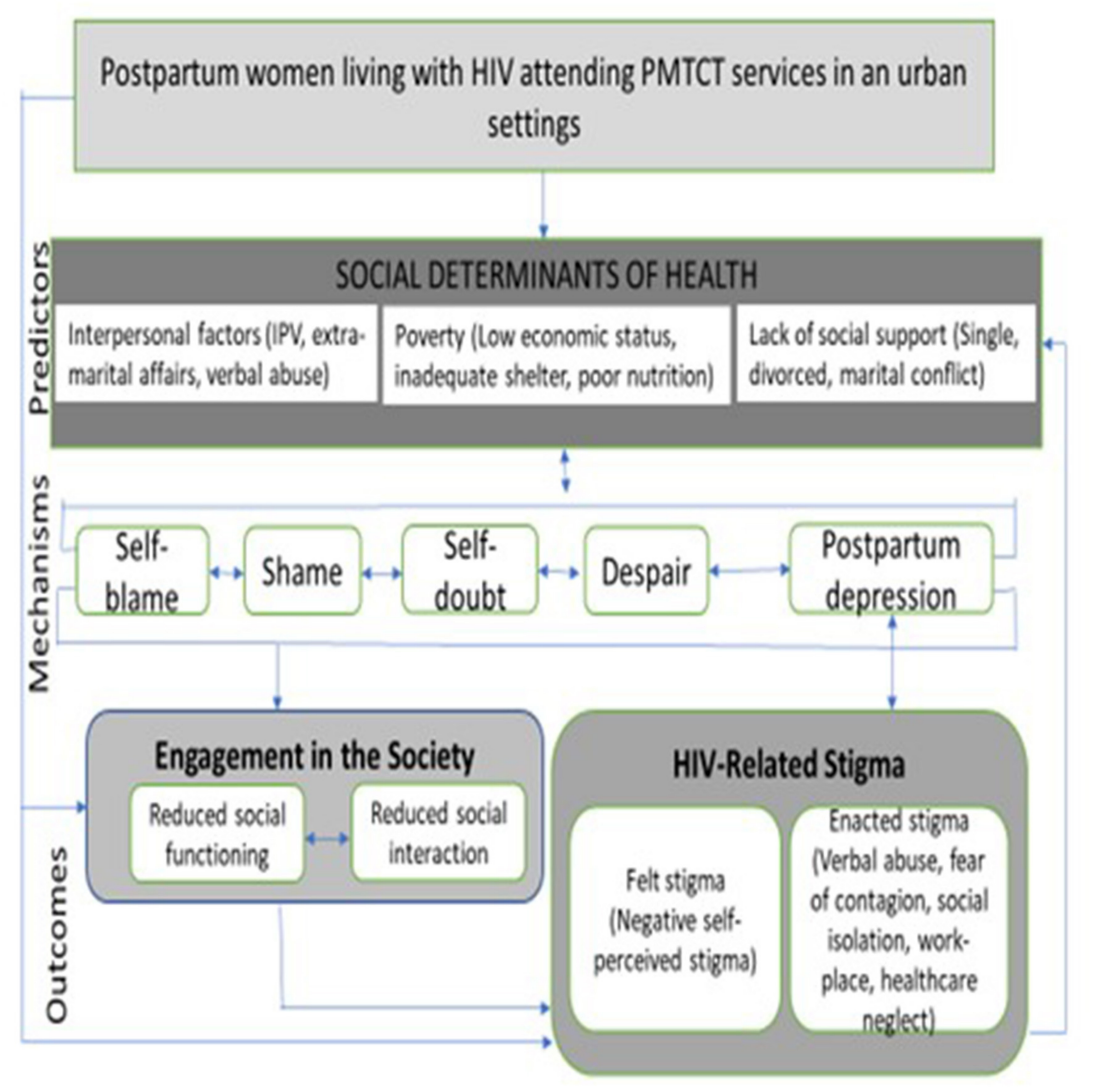
Conceptual framework for HIV-related stigma (Adapted from (32)).

Our interest is to understand HIV-related stigma (internalized and enacted stigma). We sought to understand some of the individual and interpersonal level determinants of mental health such as -intimate partner violence (IPV), partner extra-marital affairs, verbal abuse; poverty or a low economic status, inadequate shelter, poor nutrition, and lack of social support including either being single, divorced, or having marital conflicts that influence how people react to the challenges of life including self-blame, shame, self-doubt, and despair. Although borrowed from Turan's model of mechanism associated with intersectional stigma, we do feel that elements of the model inform our thinking on postpartum depression and stigma in the current work (32). Persistent manifestation of these self-perceived deficiencies will alter how one interacts or engages with others in the society (reduced social functioning, reduced social interaction) thus informing on the forms and levels of HIV-related stigma (See Figure 1). These altogether impact depression that occurs during the postpartum context and the intensity of these factors modulate the severity of depression experienced.

## Data Analysis

Descriptive statistics for socio-demographic variables, depression, and HIV-related stigma scores were computed. HIV-related stigma means, median, and SD were reported as the outcome scores. We generated data on HIV-related stigma, internalized and enacted, to assess the prevalence and associated risks with each stigma type. Some of the stigma subscales (health care neglect and workplace stigma) were less relevant to our population so this collapsing of the categories into internalized and enacted were considered important. Both Pearson's correlation for continuous variables and Spearman's correlation for categorical variables were used to determine factors that were associated with HIV-related stigma variables at the bivariate level. Variables that were found to be associated with HIV-related stigma at a significant level of *P* < 0.05 were entered into the multivariate analysis using generalized linear models (GLM) with identity links. Negative coefficients were interpreted as being protective factors of stigma whereas positive ones were interpreted as risk factors of stigma. All tests were two sided with a statistical threshold set at *P* < 0.05. All the statistical analyses were conducted using SPSS version 23. In-depth interviews were conducted among the six participants to understand their perceptions and experiences while living with HIV. Emerging themes were identified with a view to explore psychological issues associated with HIV-related stigma. We chose to identify women with high depression vs. low depression scores to better understand articulation of their HIV related life experiences, stigma, and general psychological well-being.

## Results

### Socio-Demographic and Other Characteristics of Respondents

Table 1 presents the socio-demographic characteristics of the respondents. A total of 123 HIV infected postpartum women were enrolled in the study. The majority of the women were married (68.3%), with a median age of 32 years (IQR). About 23 participants (18.7%) of our sample had completed primary school education or below, 38 participants (30.9%) had completed secondary school education, and 62 participants (50.4%) had attained an education level of college and above. About 41 (33.3%) participants were unemployed at the time; average income among those employed was 100 USD. More than half of our participants, about 69 (56.1%), reported having no social support from the family. Fifty-nine (48%) women screened positive for significant depressive symptoms.

**Table 1:** Social demographic, psychosocial and healthcare characteristics of the respondents.

**Variable**	**Category**	**Frequency** **(*N* = 123)**	**Percent** **(%)**
Marital status	Lives without male partner	39	31.7
	Married	84	68.3
Age	(Mean; Median; SD; Range)	(31.2; 32.0; 5.2; 19–48)	
Occupation	Unemployed	41	33.3
	Employed	82	66.7
Education level	Primary and below	23	18.7
	Secondary	38	30.9
	College and above	62	50.4
Income (USD)	<100 USD	68	55.3
	100 USD and above	55	44.7
Family Social Support	No	69	56.1
	Yes	54	43.9
Experience of Intimate Partner Violence	No	77	62.6
	Yes	30	24.4
	*Missing*	16	13.0
Partner engaging in Extra marital affairs	No	81	65.9
	Yes	25	20.3
	*Missing*	17	13.8
Have been treated with STI in the past 1 month	No	111	90.2
	Yes	12	9.8
Persons residing with the participant	Alone	6	4.9
	Others	117	95.1
**Clinical outcome on PPD and stigma**			
Post-Partum depression	Normal	64	52.0
	Probable major depression Probable Minor depression	37 22	30.0 18.0

### Stigma and Various Subtypes

**Figure 2:**
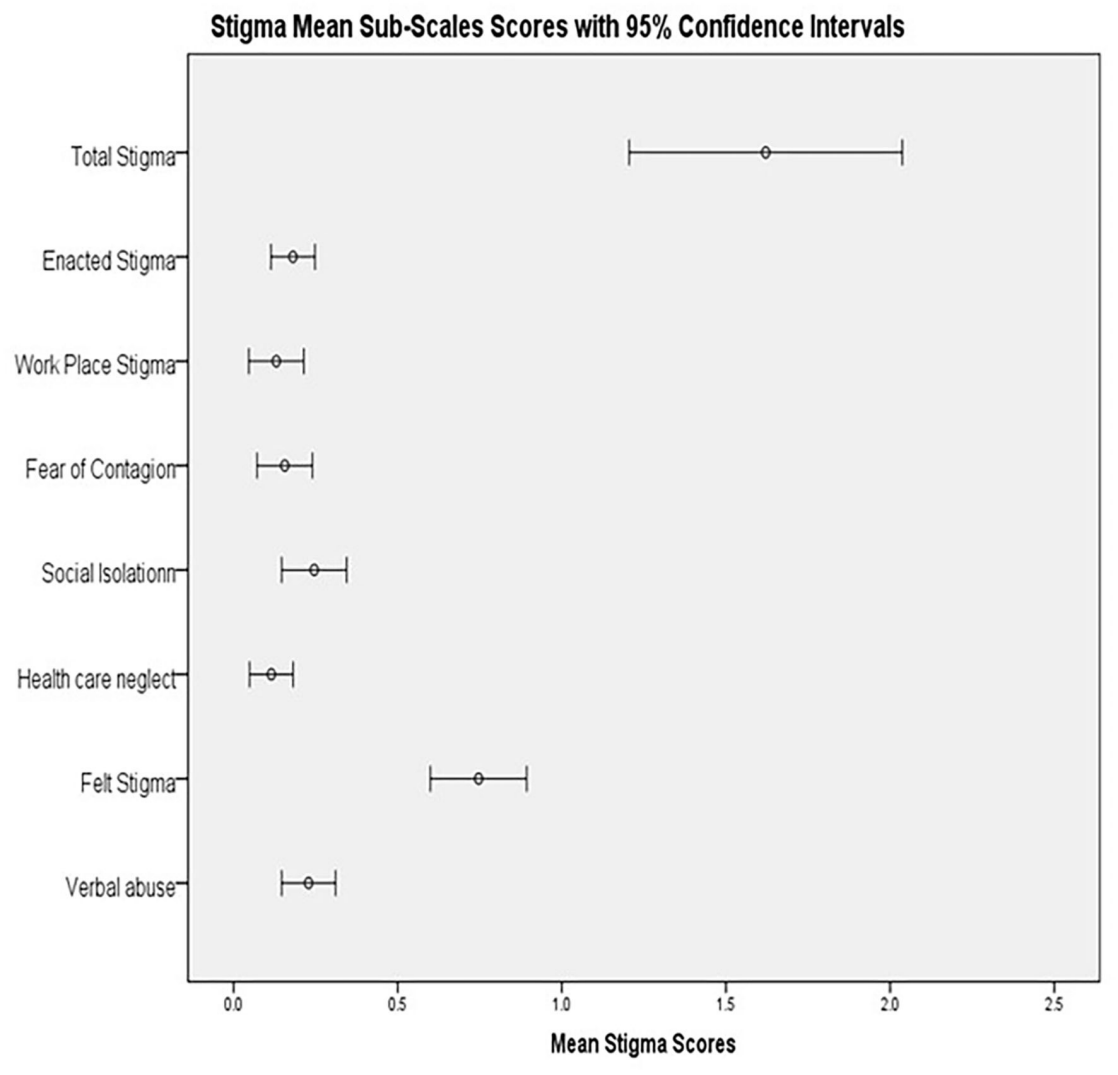
Mean subscales HIV-related stigma scores with 95% CI.

Table 2, Figure 2 present the reliability and descriptive statistics of the HASI-P scale. The reliability of different sub-scales ranged from 0.780 (fear of contagion) to 0.902 (workplace stigma). Participants' internalized stigma ranged from a score of 0–3. Participants' mean internalized stigma score was 0.75 (SD = 0.40), mean enacted stigma score was 0.18 (SD = 0.03) and, mean total stigma score was 0.27 (SD = 0.39).

**Table 2:** Reliability scores and descriptives of the stigma subscales.

**Scale**	**No. of Items**	**Reliability** **(Cronbach's α)**	**Mean(SD)**	**95% C.I**	**Median**	**Range**	**IQR**
Verbal abuse	8	0.856	0.23 (0.46)	0.15–0.31	0.00	0.00–2.63	0.13
Negative self–perception	5	0.857	0.75 (0.40)	0.60–0.89	0.89	0.00–3.00	1.40
Health care neglect	7	0.893	0.11 (0.37)	0.05–0.18	0.00	0.00–2.00	0.00
Social isolation	5	0.862	0.25 (0.55)	0.15–0.34	0.00	0.00–2.40	0.00
Work place stigma	6	0.902	0.16 (0.47)	0.07–0.24	0.00	0.00–2.83	0.00
Fear of contagion	2	0.78	0.13 (0.47)	0.05–0.21	0.00	0.00–2.50	0.00
**Enacted stigma**^**^†^**^	n/a	n/a	0.18 (0.03)	0.11–0.25	0.12	0.00–2.00	0.18
**Overall stigma**^**^‡^**^			0.27 (0.39)	0.20–0.34	0.12	0.00–2.00	0.33

† *Mean of five scales excluding negative self-perception constitute the enacted stigma*.

‡ *Mean of all the subscales constitute the overall stigma; IQR-Interquartile Range*.

*The enacted stigma signifies a combination of the four subscales (Verbal abuse, health care neglect, social isolation, work place stigma and fear of contagion). The overall stigma signifies a represents both Enacted and felt stigma*.

### Correlation Analyses Results

Table 3 presents the correlation between HIV-related stigma and other outcomes. There were statistically significant positive correlations *(P *<* 0.05)* between internalized stigma and PPD *(r* = *0.43)*, internalized stigma and marital status *(r* = *0.19)*, enacted stigma and PPD (*r* = 0.37), enacted stigma and treated for STI *(r* = *0.24)*, and total stigma and PPD *(r* = *0.50)*. Negative correlations with internalized stigma were found for age *(r* = −*0.28)*, social support (*r* = –0.26), and family income *(r* = −*0.22)*. Negative correlations were also found with enacted stigma on education *(r* = −*0.34)*, and income *(r* = −*0.20)*, and with total stigma on education *(r* = −*0.31)*, income *(r* = −*0.31)*, and social support *(r* = −*0.27)*.

**Table 3:** Correlations between HIV stigma and socio-demographic and other characteristics of the participant's (*N* = 123).

**Spearman's correlation**	**1**	**2**	**3**	**4**	**5**	**6**	**7**	**8**	**9**	**10**	**11**	**12**	**13**	**14**
Internalized stigma	1													
Enacted stigma	0.35^**^	1												
Overall stigma	0.83^**^	0.75^**^	1											
Postpartum depression	0.43^**^	0.37^**^	0.50^**^	1										
Age in years^**^‡^**^	−0.28^**^	−0.08	−0.16	0.01	1									
Marital status	0.19^*^	−0.13	−0.01	0.06	0.05	1								
Occupation	−0.05	−0.04	−0.06	0.02	0.15	−0.04	1							
Education level	−0.16	−0.34^**^	−0.31^**^	−0.32^**^	0.10	0.07	0.18^*^	1						
Income per month	−0.22^*^	−0.20^*^	−0.31^**^	−0.11	0.23^*^	0.02	0.39^**^	0.29^**^	1					
Social Support by Family	−0.26^**^	−0.16	−0.27^**^	−0.23^*^	0.04	0.04	−0.03	0.13	0.00	1				
Spouse Abuse	0.08	0.09	0.09	0.03	−0.05	−0.01	0.06	0.11	−0.04	−0.22^*^	1			
Engaging in Extramarital affairs	−0.08	−0.04	−0.08	0.14	−0.10	0.05	0.04	−0.02	−0.03	−0.11	0.34^**^	1		
Treated with STI	0.13	0.24^**^	0.15	0.07	−0.17	0.05	0.06	−0.02	−0.02	−0.07	0.13	0.18	1	
Persons Living With	0.04	0.06	0.04	−0.01	−0.05	0.09	0.08	−0.03	−0.02	0.05	−0.10	0.11	−0.05	1

** *Correlation is significant at the 0.01 level (2–tailed)*:

‡ *Pearson's correlation*.

* *Correlation is significant at the 0.05 level (2-tailed). The bold values depicts the significant correlations between the variables*.

### Multivariate Analyses Using Generalized Linear Model (GLM)

Table 4 presents the independent predictors of stigma after controlling for other factors.

**Table 4:** Multivariate generalized linear models of factors associated with HIV stigma (*N* = 123).

**Variable**	**Category**	**Internalized stigma** ***Model 1***			**Enacted stigma** ***Model 2***			**Overall stigma** ***Model 3***		
		**β (S.e)**	**95% C.I**	**Sig**.	**β (S.e)**	**95% C.I**	**Sig**.	**β (S.e)**	**95% C.I**	**Sig**.
Postpartum depression	Yes	0.64 (0.13)	0.38–0.89	** <0.001**	0.14 (0.06)	0.02–0.26	**0.018**	0.22 (0.06)	0.10–0.34	** <0.001**
	No	Ref.			Ref.			Ref.		
Age in years	Years	−0.04 (0.01)	−0.06 to −0.01	**0.002**	0.00 (0.01)	−0.01 to 0.01	0.522	0.00 (0.01)	−0.01 to 0.01	0.662
Marital status	Married	0.27 (0.13)	0.01–0.54	**0.041**	−0.12 (0.06)	−0.24 to 0.00	0.060	−0.06 (0.06)	−0.18 to 0.06	0.345
	Single	Ref.			Ref.			Ref.		
Education level	College and above	0.15 (0.18)	−0.20 to 0.50	0.400	−0.18 (0.08)	−0.35 to −0.02	0.029	−0.13 (0.08)	−0.30 to 0.03	0.112
	Secondary	0.16 (0.19)	−0.20 to 0.53	0.387	−0.08 (0.09)	−0.25 to 0.10	0.393	−0.04 (0.09)	−0.21 to 0.13	0.652
	Primary and below	Ref.			Ref.			Ref.		
Income per month	10, 000 and above	−0.25 (0.13)	−0.50 to 0.00	0.049	−0.14 (0.06)	−0.26 to −0.03	**0.017**	−0.16 (0.06)	−0.28 to −0.04	**0.008**
	<10,000	Ref.			Ref.			Ref.		
Social Support	Yes	−0.31 (0.12)	−0.55 to −0.06	**0.014**	−0.05 (0.06)	−0.16 to 0.07	0.411	−0.09 (0.06)	−0.20 to 0.03	0.136
	No	Ref.			Ref.			Ref.		
Treated with STI	Yes	0.15 (0.20)	−0.25 to 0.55	0.449	0.41 (0.10)	0.22–0.60	** <0.001**	0.37 (0.10)	0.18–0.56	** <0.001**
	No	Ref.			Ref.			Ref.		

*The bold values depicts factors that were significantly associated with the outcome variables at significant level of p <0.005*.

### Internalized Stigma

Participants who were older (β = –0.04, *p* = *0.002, 95% CI: –*0.06 to –0.01) and received social support from family members (β = 0.31, *p* = *0.014, 95% CI:* −*0.55 to* −*0.06*) experienced significantly lower levels of internalized stigma, as compared to those who were young (older age was associated with less stigma; each year increase in age was associated with 4% lower odds of internalized stigma) and those who lacked social support.

Participants who had postpartum depression (β = 0.64, *P* <* 0.001, 95% CI*:0.38–0.89) and those who are married (β = 0.27, *p* = *0.041, 95% CI*:0.01–0.54) had significantly higher levels of internalized stigma as compared to those who did not have postpartum depression and those who are single, divorced, separated, or widowed.

### Enacted Stigma

Participants who had PPD (β = 0.14, *p* = *0.018, 95% CI*:0.02–0.26) and those who have been treated for STIs (β = 0.41, *P* <* 0.001, 95% CI*:0.22–0.60) had significantly higher levels of enacted stigma, as compared to those who did not have PPD and those who had never been treated for STI.

Participants who earned 100 USD and above per month (β = –0.14, *p* = *0.017, 95% CI: –*0.26 to –0.03) experienced significantly lower levels of enacted stigma, as compared to those who had an earned income of <100 USD per month. Participants who had a college education and above had significantly lower levels of enacted stigma (β = –0.18, *p* = *0.029, 95% CI: –*0.35 to –0.02) as compared to those with a primary school education or below.

### Overall Stigma

Participants who had PPD (β = 0.22, *P *<* 0.01, 95% CI*:0.10–0.34) and have been treated for STIs (β = 0.37, *P *<* 0.001, 95% CI*:0.18–0.56) had significantly higher levels of total stigma, as compared to those who did not have PPD and those who had never been treated for STIs. Participants who earned 100 USD and above per month (β = –0.16, *p* = *0.008, 95% CI: –*0.28 to –0.04) experienced significantly lower levels of total stigma, as compared to those who had an earned income of <100 USD per month.

### Experiences and Perceptions of Post-partum Women Living With HIV

We offer some observations from the interviews we conducted at the end of the survey.

#### Barriers to Improved Psychological Well-Being While Living With HIV

Lack of social support, verbal abuse, and poverty seems to lower their ability to cope with their HIV status as shown by participants who had scored >20 on EPDS. Some male partners who verbally abuse their female partners could further aggravate HIV-related stigma. One of the respondents, Beatrice (a pseudonym) who was diagnosed with significant depressive symptoms explained how this could worsen the situation.:

“I got pregnant while in form 3 when I was of age 18 years and I have always regretted that day. My husband continues to beat me and telling me to go away with allegations of having infected him with HIV which pains me a lot since I know I had no other friend since I got married to him. My neighbor in a rented house told me ‘why are you coughing too much, you could go to be tested or you have HIV already?’ This made me feel like dying since I thought they had been discussing me with other women in the neighborhood.”

Diana (a pseudonym) who has significant depressive symptoms could actually demonstrate how her poor family background made her more vulnerable to PPD and HIV-related stigma:

*“I dropped out of form two* (*2*) *in the year 1998 due to lack of school fees and stayed at home. In the year 2007 I got pregnant to a man and we got married and since then he has taken me through hell. At the moment, I only believe prayers could help. If God can touch my husband to avoid verbal abuse and assist in financial support, life could be better. Also, I wish God could provide me with some work to do for a living even if washing clothes for pay.”*

Lack of social support from the male partner was also noted to affect their quality of life. Rachel (not her real name), who has significant depressive symptoms shared the following:

“I lack sleep mostly when I am angry with my husband. I cry because of life challenges without good social support. I have thought of separating from him but where will I go. I don't have parents. I think those with parents are lucky since most of them will never get tired of listening and helping them. All my remaining brothers are alcoholic and never got married hence I get no support from them.”

#### Facilitators to Improved Psychological Well-Being While Living With HIV

Those participants with EPDS <13 narrated how they are coping fairly well with their daily living in the society. Jacquie (a pseudonym), who did not have significant depressive in her testimony affirms the success of the PMTCT program at KNH:

“I am a mother of two children and through PMTCT efforts, my last born of the year 2012 has tested negative for HIV. I think of my kids and I get the will power to live and care for them further. I have shared my status with one of my brothers, my mother and also my husband are aware. My friends who are infected should find someone talk to. Like for me I share with my mum and crying and expressing oneself also helps.”

Ester (a pseudonym) who did not have significant depressive symptoms in the EPDS pointed out an important realization about the value of education and its connection with livelihood:

“My advice to others is that if they can afford to go back to school, they should pursue education. Currently I am working as a cleaner at a local secondary school which is very unreliable job. I have worked there since the year 2009 and still struggling to survive.”

In our study, Pamela (not her real name), who did not have significant depressive symptoms appeared to have a great insight into the value of social support and had this to say:

“Those with this disease (HIV) should make themselves busy with their daily work, avoid anger, accept one's status, take medication, share concerns with their husbands or close friends they trust. Also, one should create time to visit the affected and vulnerable children since it makes one feel good for helping others.”

## Discussion

### Socio-Demographic and Other Characteristics of Respondents

In our findings, 68.3% of the women living with HIV were married and this figure is lower than some other studies from Kenya which reported 96% (33), and with a 79.7% (34) marriage rate amongst their participants. The lower rate of those who are married in our study population could be due to the fact that they had better education and therefore were made up of more independent urban women. It has been found that higher educational levels are associated with a single or divorced status in urban settings with changes in cultural values among Kenyan women (35). In addition, 50.4% of our participants had educational levels above secondary school level education which was higher than the study carried out in the Kibera slum (25.4%) where educational levels were significantly lower. In Kenya, PPD for women within the general population has been reported to be 18.7% (36) and a systematic review in Ethiopia found PPD to be at 22.89% (37) with another one in the same country reporting a prevalence of 23.7% (38). The higher PPD for this study population could be due to the greater challenges faced by people living with HIV in general as well as depression along with treatment fatigue being known side-effects of ART.

### Internalized Stigma: Contributor to the Depression Pathogenesis

Our study highlights that postpartum depression is significantly associated with internalized stigma. Internalized stigma impacts people's daily lives, it affects the way in which they cope with their HIV-positive status and how they behave socially (39). Being of older age, having an income above 100 USD per month, and good family social support were found to be positively associated and may potentially be protective factors safeguarding individuals from internalized stigma. However, most of our study participants had an income below 100 USD per month which increased the odds of additional life adversities including health care burden, as supported by findings of a previous study where up to 48% of low-income mothers reported elevated postpartum depression symptoms (40).

People living with HIV perceive the negative stereotypes to be legitimate and suffer negative cognitive, emotional, and behavioral consequences such as ambivalence about identity, low-self-esteem, and low self-efficacy (20, 41). Lack of family and social support was seen as a trigger for negative self-perceived stigma. Recent studies have shown that PLWH, who report experiences of HIV-related stigma also report lower levels of perceived social support (22). Social support refers to the provision of psychological and material resources by people within one's social network (42). At the individual level, interventions should be focused on enhancing social support by activating or strengthening existing ties (43). Our study's findings concur with a South African study where HIV internalized negative attitudes perceived to be associated with HIV and resulted in feelings of low self-worth which became strong predictors of PPD among women living with HIV (18). Negative self-perception is an internalized stigma that perpetuates and feeds into depression (with key cognitions being feelings of shame, self-denial, guilt, secrecy, no disclosure, and despair) and needs to be addressed through support groups and individual psychotherapy work.

### Enacted Stigma and Its Association With Depression

Postpartum depression was also found to be closely associated with enacted stigma which is consistent with previous studies where a high level of HIV-related stigma has been strongly associated with a high level of depression and a low level of self-efficacy (8). Similar findings from a systematic review including those of a study from Uganda, demonstrated a strong association between HIV-related stigma and PPD among PLWH even in the general population (44). Our findings are consistent with other previous studies that showed thar HIV-related stigma can manifest in social isolation (45, 46). Enacted stigma presents in the form of blaming, judging, insulting, and name calling which also featured in our in-depth-interviews with some of the participants (47).

### Association of Overall Stigma With PPD

Overall, postpartum depression was considerably associated with internalized stigma, enacted stigma, and total stigma scores. As in this study, PLWH, who report experiences of HIV-related stigma also report low levels of perceived social support (22). Overall, we found that our participants with more severe depressive symptoms had high levels of stigma (β = −2.65, *p* = *0.001, CI:* −3.93 to −1.37). We did find a significant relationship between stigma and depressive symptoms similar to a study conducted in Kenya (17) (see Figure 2). HIV-related stigma has been well-documented to negatively impact quality of life and overall health outcomes among persons living with HIV (15). Our findings also concur with a study from Canada where a higher level of HIV-related stigma was strongly associated with a higher level of depression, accompanied with poor self-efficacy (8).

### Perpetuation of Psychological Distress Emanating From Both Types of Stigma

Internalized and enacted stigma association clearly demonstrates that persons living with HIV experience numerous mental and psychological sequela of stigma, including stress, fear, anxiety, decreased self-esteem, and depression (7, 9). HIV remains a complex concept associated with blame, shame, disgrace, and social unacceptability (48).

**Figure 3:**
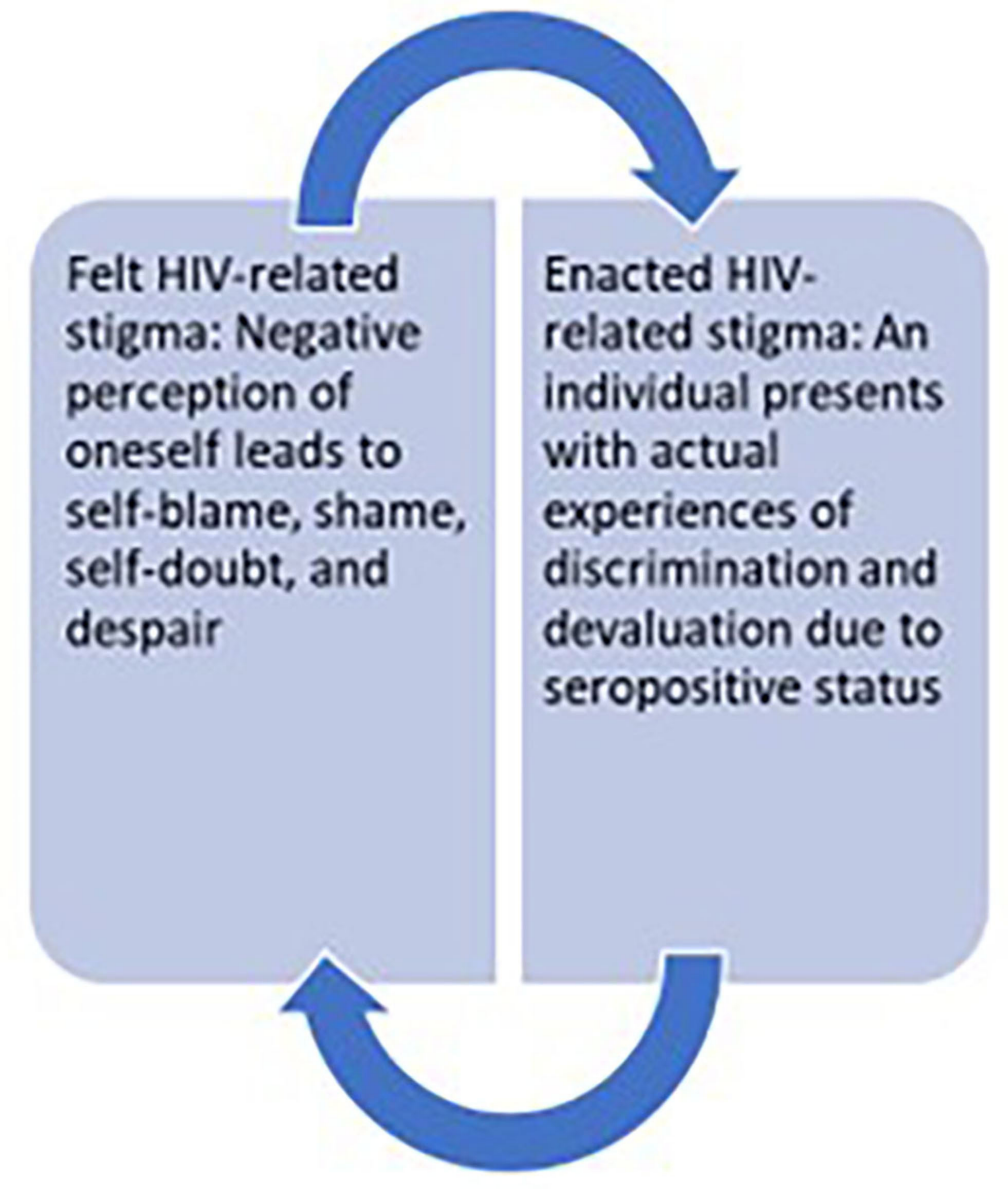
Perpetuation of HIV-related stigma and psychological distress.

In our study, social isolation was the most common form of enacted stigma (See Figure 2). However, in our study we internalized that more than the enacted stigma, certain social determinants of health such as poverty, intimate partner violence, and spousal extramarital relationships added more vulnerability in the lives of our participants as found during the in-depth interviews. HIV-related stigma may instill shame in people living with HIV with psychological torture arising from persistent negative feelings toward oneself (49) (also see Figure 3). Generally, pregnant women living with HIV experience a lot HIV-related stigma and discrimination which predisposes them to depressing and suicidal thoughts, most of the time within the society (13). In our study, in-depth interviews revealed lower perceived social support from their spouses which has been reported as one of the things that poses challenges to people living with HIV (50).

### Prioritizing Stigma Reduction and Mental Health in Future HIV Prevention and Care

Previous studies concur with findings reported in this study which show that HIV-related stigma is a strong predictor of PPD among HIV-positive women (18). Negative self-perceived stigma has been predominant in other studies such as the one carried out in the rural Nyanza province of Kenya (16). High prevalence of negative self-perceived stigma was also reported in a Ugandan study where participants described themselves as “useless” and “same as dead” (14). Social support is a critical effect modifier in addressing HIV-related stigma as reported in one of the developed countries (51). Meaningful interventions should target spousal or familial support as being critical to enabling persons living with HIV to overcome enactments of HIV-related stigma and other obstacles to care, and to successfully adhere to treatment (52, 53). We recommend an integrated care approach, where mental health services could be embedded within the PMTCT clinic to screen and diagnose early features of mental illness, to mitigate various psychosocial risks, and to offer curative and preventative health care.

## Limitations

Our study was not without its limitations. Our data did not capture information relating to the family structure and living conditions of the participants. Possible sources of enacted stigma may therefore not be clearly defined to a specific variable which might have been valuable in further interpreting our findings. We used EPDS, which is a screening tool and not a formal clinical assessment, in the service of time and ease of assessment. We began this research as a primary focus on PPD and HIV-related stigma as a secondary risk factor, therefore, our focus may have been regimented and not open enough to understand other factors that may impact HIV-related stigma more directly. Items used to measure both constructs of both internalized stigma and depression had closely related symptoms. The study was somewhat underpowered, and this might have caused some variability in results. Future research should explore issues with male partner involvement in the PMTCT program and assessment of community perception toward persons living with HIV, which we were not able to ascertain in this study. We carried out a limited qualitative exploration and future studies might want to use more intensive mixed-methods to understand participant stigma and depression experiences.

## Conclusion

From our findings, HIV-related stigma burden and postpartum depression in women attending PMTCT needs urgent redress and health services' attention. Future interventions should be aimed at empowering persons living with HIV with life skills and depression care that will improve their quality of life and the well-being of their baby.

We also recommend interventions involving spousal and family support. PMTCT is the key to safe motherhood and child health outcomes in this subgroup of women and in this care cascade; measures have to be developed to enhance mental health including mitigating PPD.

## Data Availability Statement

The datasets generated for this study are available on request to the corresponding author.

## Ethics Statement

The studies involving human participants were reviewed and approved by Ethical approval was obtained from the Kenyatta National Hospital/University of Nairobi Ethical and Research Committee (KNH/UoN-ERC) Ref. no. P171/03/2014. The patients/participants provided their written informed consent to participate in this study.

## Author Contributions

The work was carried out by OY as part of the Masters' degree in clinical psychology at the department of Psychiatry University of Nairobi. OY collected data and wrote the findings. MK was the primary mentor and helped in conceptualizing, writing, and conducting statistical analysis. MM was the second supervisor who assisted during planning of the research concept and reviewed the results. TA analyzed and interpreted the results. All authors read and approved the manuscript.

## Conflict of Interest

The authors declare that the research was conducted in the absence of any commercial or financial relationships that could be construed as a potential conflict of interest.

## Abbreviations

AIDS: Acquired Immunodeficiency Syndrome
ART: Antiretroviral Therapy
ARV: Antiretroviral Drugs
EPDS: Edinburgh Postnatal Depression Scale
GLMs: Generalized Linear Models
HIV: Human Immunodeficiency Virus
IPT-G: Group Interpersonal Psychotherapy
KNH: Kenyatta National Hospital
LMIC: Low- and Middle-Income Countries
PLWHAs: Persons Living with HIV/AIDS
PMTCT: Prevention of Mother-to- Child Transmission
PPD: Postpartum Depression
SD: Standard Deviation
SPSS: Statistical package for the social sciences
STDGs: Sustainable Development Goals
WHO: World Health Organization.
